# The fight for evolution

**DOI:** 10.7554/eLife.84343

**Published:** 2022-11-09

**Authors:** N Ezgi Altınışık

**Affiliations:** 1 Department of Anthropology, Hacettepe University Ankara Turkey

**Keywords:** sparks of change, science, politics, turkey, theory of evolution, resistance, research culture

## Abstract

As the Turkish government intensified its attacks on the theory of evolution, the academic community rallied to push back. A researcher recounts how she decided to join them.

My story begins in 2007, the year I started to study molecular biology and genetics at Istanbul University; the year which also saw the deeply conservative Justice and Development Party, or AKP, win the Turkish general elections for the second time in a row. I was excited to finally get into university and explore different fields of biology. Soon, however, pressure on secular people and institutions started to increase, with censorship in the media and on the Internet becoming palpable. Anti-evolution sentiment was on the rise, but while the situation looked bleak, many inside and outside universities continued to hope.

After all, Turkey has a long history of political opposition to the theory of evolution. Darwinian concepts arrived in the region in the late 19^th^ century and were widely taught in school as early as the 1920s. But the idea of change, which is the basis of evolution, often conflicts with conservative ideologies. In the 1970s, representatives of conservatives in the parliament attempted to have the theory of evolution removed from the curriculum. Left-wing movements and teachers’ unions stood in their way until a military coup disbanded all political organisations a few years later.

From then on, anti-evolutionist ideas became embedded in state policy. Influential groups established close bonds with the Creation Research Institute in the United States and creationism found its way into textbooks in the mid-1980s. Cult leader Harun Yahya came to the fore during the following decade, leading a well-funded campaign to spread ‘intelligent design’ concepts at home and abroad. The situation further hardened in the early 2000s when the AKP won its first elections. Pro-governmental organisations started to wage war against evolutionary biology, using fake fossils, misleading pamphlets and books full of lies to spread their ideas in schools and universities. I had been taught creationist ideas in high school, but I had not fully realised their political importance at the time.

For me, the breaking point came in 2009. To mark the 200th anniversary of Darwin’s birth, the science magazine Bilim ve Teknik decided to dedicate its front cover and several articles to the famous naturalist. The government banned the issue: the cover was changed, the articles were removed, and the editor-in-chief (one of Turkey’s leading archaeologists) was fired. I still remember my outrage when I heard the news. Bilim ve Teknik is run by TÜBİTAK, a state agency that grants scientific funding. For a long time, it was the only science magazine widely accessible in Turkey. Many people in my generation, myself included, first encountered science through its pages. Massive demonstrations were held across the country in solidarity with the editor. My friends and I visited every professor in our department, encouraging them to join the protest outside of our university. Thousands of young people eagerly attended events that encouraged the defence of the theory of evolution. It was so exciting to see.

The protests also made me think about how I could help to fight obscurantism. For me, only organised resistance could defeat organised oppression. Others were already trying to spread scientific facts about evolution through blogs, conferences and seminars. Under the name Evrim Çalışkanları (’hard-workers for evolution’), graduate students around the world were setting out to translate into Turkish the website Understanding Evolution (evolution.berkeley.edu), which is run by the University of California, Berkeley. At home, a student society was organising an annual meeting dedicated to the theory of evolution. Later on, this would become the Aykut Kence Evolution Conference, named in honour of a leading Turkish evolutionary biologist.

By 2011, I had become sure that I wanted to study evolutionary biology; this is when I was invited to join evoeko, a mailing list which allowed Turkish evolutionary biologists around the globe to come together in solidarity. Like many of my peers, my future would be shaped by this community and the guidance we received from senior members. Together we discussed academic questions and the best ways to resist political interference in biological sciences. We started to organise conferences for teachers, students and the general public, simply to teach evolution.

**Figure 1:**
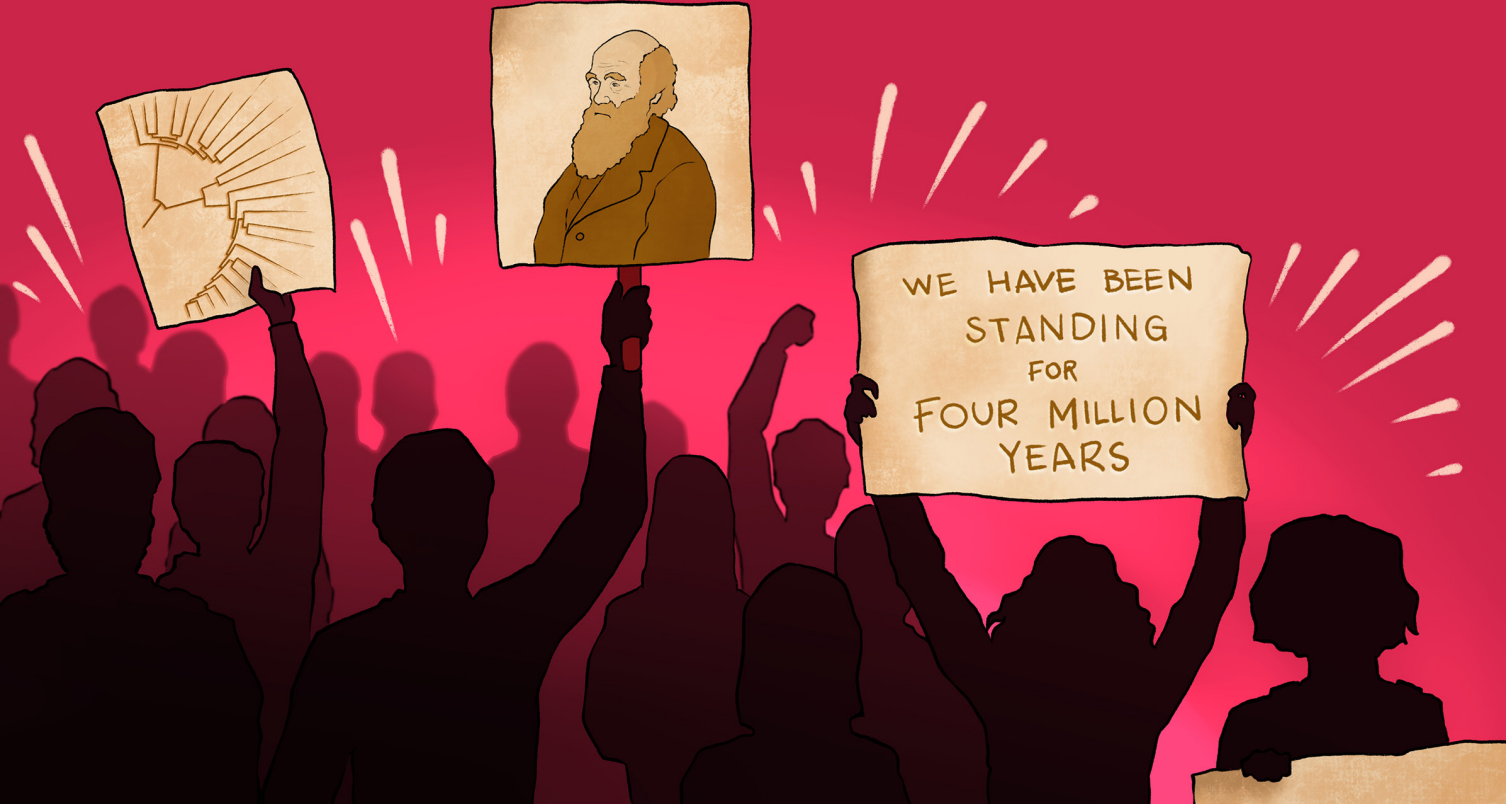
Protestors held signs defending the theory of evolution during demonstrations against the Turkish government.

Image credit: Marzia Munafo; @munafomarzia (CC BY 4.0)

A wave of contestation swept through the country in 2013, starting in Gezi Park, Istanbul and quickly spreading to all cities in Turkey. People stood up against oppression with demands for freedom. In places such as Gezi Park, where peaceful protestors managed to resist police attacks, forums were organised to cover social and scientific topics including evolution. At demonstrations, people held signs reading “4 milyon yıldır ayaktayız”; “We have been standing for four million years”. The protests were crushed, yet many people carried on holding these forums in their neighbourhoods. The next year we launched the annual Ecology and Evolutionary Biology Symposium, the first academic event in this field to take place in Turkey; in 2015, we established our country’s first Ecology and Evolutionary Biology Society.

I also started my PhD that year – on the population history of the Beringian and North American arctic region – and relocated to the University of Ostrava in Czechia. One day, I heard that my lab mate had seen a lavish book promoting creationism in the institute. I was surprised and embarrassed to learn that a group in Turkey had sent it, to try to spread their fake arguments abroad. At home, the government finally announced that the theory of evolution would be removed from the high school curriculum. Still, I decided to return to Turkey after my PhD. Many people tried to stop me, except colleagues from the evolutionary biology community who were leading the resistance at home. I just couldn’t ignore what was happening in my country, close the door of the lab, and focus solely on my research.

Of course, this decision has come with challenges. Turkish science budgets are limited, political opponents are being silenced, and an ongoing economic crisis is tightening its grip. Yet our research and our outreach work are going forward. I am conducting ancient DNA studies from an evolutionary perspective; my group includes several evoeko members, as well as the former editor-in-chief of Bilim ve Teknik. Our work is funded by the European Research Council through a senior group member, but I have recently received my first-ever research grant from TÜBİTAK to explore population history in Roman era.

As we dream of a better country, we continue to resist. Following meetings at the ministerial level, board members of the Society have managed to get some basic evolutionary concepts reinstated to the curriculum. Volunteers have been organising the Aykut Kence Evolution Conference for over 16 years now, passing it on from one generation of students to the next. It attracts over a thousand attendees every year; when they invited me as speaker, I was amazed by the ambitions of those in attendance. Together with my peers, I still join and organise online and on-site events to promote scientific thinking and enlightenment to students and the public – for example, an online series on human evolution has already received several thousand views and is still getting attention. We also do not limit ourselves to evolutionary biology anymore. As in other parts of the world, anti-vaccine movements rose in Turkey during the pandemic, aided by the recent decline in basic science education. Communicating scientific thinking is more important now than ever.

As a scientist, I believe I have a responsibility towards the people whose taxes funded my education and now fund my research. I am indebted to those who have guided me in the dark as a young student, and to those who cherish the dream of becoming a scientist in Turkey one day. I cannot say that our careers as evolutionary researchers have all been easy, but they may not have been as difficult as one could think. My journey has taught me that when oppressed people stop being alone, they also stop being afraid. To those who need hope and believe in the idea of change, you are not on your own. Our stories will also be your story.

## Share your experiences

This article is a Sparks of Change column, where people around the world share moments that illustrate how research culture is or should be changing. Have an interesting story to tell? See what we’re looking for and the best ways to get in touch here.

